# Pain level between clear aligners and fixed appliances: a systematic review

**DOI:** 10.1186/s40510-019-0303-z

**Published:** 2020-01-20

**Authors:** Paula Coutinho Cardoso, Daybelis Gonzalez Espinosa, Paulo Mecenas, Carlos Flores-Mir, David Normando

**Affiliations:** 1 Federal University of Pará (UFPA), Belém, Pará Brazil; 2 Faculty of Medicine and Dentistry, University of Alberta, Edmonton, Canada; 3 Faculty of Dentistry, Federal University of Pará (UFPA), Rua Augusto Correa 01, Belém, Pará 66075-110 Brazil

**Keywords:** Orthodontic appliances, Pain, Invisalign, Malocclusion

## Abstract

**Objectives:**

To assess if there is any difference in pain levels between orthodontic treatment with clear aligners or fixed appliances.

**Materials and methods:**

An electronic search was completed in PubMed, The Cochrane Database, Web of Science, Scopus, Lilacs, Google Scholar, Clinical Trials, and OpenGrey databases without any restrictions until February 2019. All comparative study types contrasting pain levels between clear aligners and fixed appliances were included. The risk of bias (RoB) was assessed using the Newcastle-Ottawa Scale, ROBINS-I-Tool, or ROB 2.0 according to the study design. The level of evidence was assessed through the GRADE tool.

**Results:**

After removal of duplicates, exclusion by title and abstract, and reading the full text, only seven articles were included. Five were prospective non-randomized clinical trials (CCT), one was a cross-sectional study, and one was a randomized clinical trial (RCT). Two studies presented a high RoB, three a moderate RoB, and two a low RoB (including the RCT). A meta-analysis was not performed because of clinical, statistical, and methodological heterogeneity. Most of the studies found that pain levels in patients treated with Invisalign were lower than those treated with conventional fixed appliances during the first days of treatment. Differences disappeared thereafter. No evidence was identified for other brands of clear aligners.

**Conclusions:**

Based on a moderate level of certainty, orthodontic patients treated with Invisalign appear to feel lower levels of pain than those treated with fixed appliances during the first few days of treatment. Thereafter (up to 3 months), differences were not noted. Malocclusion complexity level among included studies was mild. Pain is one of many considerations and predictability and technical outcome are more important, mainly considering that the difference does not seem to occur after the first months of the orthodontic treatment.

## Introduction

Pain is a subjective response and presents a large number of individual variations under the same trigger conditions. It depends on several factors such as age, sex, individual pain threshold, emotional state, stress, amount of applied force, cultural differences, and previous experiences of pain [1, 2]. Pain complaints are a common feature during orthodontic treatment [3] directly influencing patient’s satisfaction [4]. It is one of the main reasons for orthodontic treatment discontinuation [5].

It is well known that during orthodontic treatment with fixed appliances, it is common to feel pain and discomfort [6], reaching its peak 24 h after arch insertion, and being almost imperceptible 7 days after [7, 8]. However, the type of the appliances may have an influence on the pain and discomfort reported by the patients due to the type of force applied. Removable appliances produce intermittent forces, which allow the tissues to reorganize before compressive forces are reapplied [9]. Regarding studies that have evaluated pain levels with clear aligners compared to fixed appliances, some studies have found positive [2, 10, 11] or negativ e[12] results related to clear aligners.

When comparing quality of life (QoL) between patients treated with fixed appliances and Invisalign (Align Technology, San Jose, CA), it was observed that both presented similar QoL results, except under the category of eating and chewing where the aligner group showed better results [13].

Systematic reviews have evaluated the efficiency of orthodontic treatment with clear aligners and they suggested that the outcomes are not as accurate as those with fixed appliances [14–16]. On the other hand, treatment done with clear aligners present some advantages such as overall decreased treatment and chair time in patients with mild to moderate malocclusions [17]. Besides that, studies have shown that gingival health tends to be better, based on the periodontal health index, in patients treated with clear aligners [18, 19].

There are controversial findings regarding pain level during orthodontic treatment with fixed appliances versus clear aligners. Thus, the aim of this systematic review was to evaluate the level of pain during orthodontic treatment in patients treated with clear aligners compared with patients treated with fixed appliances.

## Material and methods

### Protocol and registration

The present systematic review was registered in the PROSPERO database (http://www.crd.york.ac.uk/PROSPERO, PROTOCOL: CRD42019131359) and was done according to the Preferred Reporting Items for Systematic Review and Meta-Analysis (PRISMA) guidelines (www.prisma-statment.org).

### Eligibility criteria

The following criteria were used in the selection of the articles:

1. Study design: Randomized or non-randomized controlled clinical trials and cross-sectional studies.
2. Population: Adult patients during orthodontic treatment.
3. Intervention: Patients treated with clear aligners.
4. Comparison: Patients treated with conventional fixed appliances.
5. Outcome: Pain level.
6. Exclusion criteria: Laboratory studies, clinical trials, case reports, literature reviews, and studies done with patients with syndromes and/or craniofacial deformities were excluded from the research.

### Information sources, search strategy, and study selection

There were no restrictions on languages and dates of publication. The search was completed by two authors (P.C.C and D.G.E.) until February 2019. The search was performed in the following databases: Cochrane, PubMed, Scopus, Google Scholar, Lilacs, Web of Science, Clinical Trials, and OpenGrey. Specific search strategies per database are shown in Appendix 1. A hand search was also performed.

The included articles were exported to a bibliography reference manager (Mendeley, version 1.19.4 Elsevier). In case of disagreement, a third evaluator (D.N) opinion was consulted.

### Data items and collection

The data collection in duplicate was carried out according to the following criteria: type of study, sample size, intervention, assessment of pain, time of evaluation, sequence of the archwires and aligners, pain outcomes, overall outcomes, analgesic consumption and author’s conclusion (Table 1).

**Table 1:** Extraction of data

Authors, (year)	Type of study (country)	Sample size, male/female ratio, and age (mean ± sd) per group (age)	Intervention	Assessment of pain	Time of evaluation	Sequence		Pain outcomes	Overall outcomes	Other outcomes	Analgesic consumption		Author’s conclusion
						Archwire	Align						
Almasoud (2018) [10]	Prospective CCT (Saudi Arabia)	CG: *n* = 32, 12M/20F (23.56 years ± 5.44) IG: *n* = 32p, 10M/22F (28.47 years ± 8.17)	CG: Passive self-ligating (Damon) IG: Invisalign	VAS 10 cm	4 h; 24 h; 3rd, and 7th day	.014″ NiTi	Firsts aligners	Patients treated with IG had significantly lower pain level than did those in CG at all timepoints. The highest pain level was 24 h	Higher numbers of participants reported having pain at 4 h and lower number in day 7	More patients in CG took analgesics when compared with the IG	Yes (acetaminophen/paracetamol)		Patients treated with Invisalign observed lower pain than did with braces. ↑of pain was experienced at 24 h and ↓ at day 7 in both groups
Flores-mir et al. (2018) [13]	Cross-sectional (Alberta, Canada)	CG: *n* = 4, NA (NA) IG: *n* = 81, NA (NA)	CG: conventional fixed appliance) IG: Invisalign	DIDL (Dental Impacts on Daily Living) PSQ (Patient Satisfaction Questionnaire)	End of treatment	NA	NA	Similar satisfaction	Eating and chewing: IG reported a better satisfaction	Patient satisfaction remained relatively similar 6 months later for the bracket-type treatment	No		Both groups treated patients had statistically similar satisfaction outcomes, except for eating and chewing
Fujiyama et al. (2014) [20]	Prospective CCT (Ohio)	CG: *n* = 55, 25M/35F (26.45 years ± 5.45) IG^1^: *n* = 38, 10M/28F (26.64 years ± 5.69) IG^2^: *n* = 52, 19M/33F (25.24 years ± 6.51)	CG: Edgewise (straight wire with .018 slot) IG^1^: Invisalign (IG) IG^2^: Edgewise + Invisalign (EIG)	VAS 10 cm	1°: 60 s, 6 h, 12 h, 1–7 days 2°: 3 weeks after 3°: 5 weeks after	Slot .018″	Use 20 h/day	EG was significantly higher than others	IG was significantly ↑ than others (intensity of pain, no. of days, discomfort)	NA	No		Invisalign causes less pain compared to the traditional edgewise treatment; problems such as tray deformation must be carefully checked in the use of Invisalign.
Mais-Damois et al. (2015)	Prospective CCT (Canada)	CG^1^: *n* = 19, NA (NA) CG^2^: *n* = 20, NA (NA) 18M/21F (14.5 years) IG: *n* = 31, 11M/20F (16 years)	CG^1^: Damon S CG^2^: Speed IG: Invisalign	VAS	0 h, 5 h, 24 h, 3rd, 7th, 14th day	- .016″ NiTi - .016″ CuNiTi - .016″ × .022″ CuNiTi - .019″ × .025″ CuNiTi		Aligners 1, 4, 7, and 10	Invisalign group reported lower pain than fixed appliances	Patients with Invisalign reported significantly less tissue irritation than patients with fixed brackets	Quality of life was slightly affected in the first phase higher in CG than in IG)	Yes Exclusively during the first week of treatment	Perception of pain with Invisalign was lower than with fixed appliance. This method of treatment is an attractive therapy for patients wishing for an esthetic treatment.
						Changed each 2 weeks							
Miller et al. (2007) [11]	Prospective CCT (USA)	CG: *n* = 27, 6M/21F (28.6 years ± 8.7) IG: *n* = 33, 11M/22F (38 years ± 12.4)	CG: preadjusted fixed appliance (NA) IG: Invisalign	- Daily diary: functional, psychosocial and pain-related (Likert Scale) - Pain (VAS)	NA	NA		NA	Fixed appliances group reported more pain beginning at day 1 through day 7	Invisalign and fixed appliances reported decreases in OHRQL after treatment beginning	The fixed appliances subjects took more pain medication during days 2 and 3	Yes	The Invisalign subjects’ overall OHRQL was better than that of the fixed appliances subjects.
Shalish et al. (2011) [12]	Prospective CCT (Israel)	CG^1^: *n* = 28, 14M/14F (NA) CG^2^: *n* = 19, 4M/15F (NA) IG: *n* = 21, 5M/16 F (NA)	CG^1^: Buccal group (straight wire GAC and Ormco) CG^2^: Lingual group (Incognito) IG: Invisalign group	HRQoL VAS (pain)	7 consecutive days and at day 14	.014″ NiTi		NA	Pain levels decreased from the 1 to 7 day. ↑ Invisalign and Lingual group	Day 1: ↑ % pain in Invisalign group; Day 2: Lingual group; Small % of buccal group reported severe pain	Oral dysfunction, disturbances in eating, general activity, recovery time: ↑ Lingual group	Yes Highest in the Lingual group (similar to the buccal group)	Lingual appliance was associated with more severe pain and analgesic consumption, the ↑ oral and general dysfunction, and the most difficult and longest recovery
White et al. (2017) [2]	RCT (USA)	CG: *n* = 18, 6M/12F (NA) IG: *n* = 23, 11M/12F (NA)	CG: Fixed clear appliance upper arch (Radiance) and metal brackets lower arch (Alexander) IG: Invisalign	VAS (10 cm)	Initial adjustment: daily diary for 7 consecutive days Subsequent adjustments (2): Daily diary for 4 days	- .016″ CuNiTi - .017″ × . 025″ CuNiTi - .016″ × .022″ SS - .017″ × .025″ SS		Change each 2 week and use 22 h/day	1°: higher in CG; 2° 3°: higher in CG	Discomfort was significantly higher in CG during the 1st week and subsequent adjustments First 3 days after bonding: more discomfort when chewing with fixed appliances.	Analgesic consumption: higher in CG in the first week; 1° and 2° adjustment no ≠ No ≠ in sleep disturbances	Yes	Traditional fixed appliances produced significantly more discomfort than did aligners. Patients treated with aligners and fixed appliances reported significantly less discomfort at subsequent adjustments than after the initial bonding or appliance delivery.

*CCT* non-randomized controlled clinical trial, *RCT* randomized clinical trial, *VAS* visual analog scale, *OHRQL* oral health-related quality of life, *NA* not available

### RoB/quality assessment in individual studies

For the cross-sectional study, the Newcastle-Ottawa Scale adapted to cross-sectional studies was used [21]. The evaluation was done by counting stars acquired in each category (Table 2).

**Table 2:** Risk of bias of the studies, according to the Newcastle-Ottawa Scale adapted for cross-sectional studies

	Selection (maximum 5 stars)	Comparability (maximum 2 stars)	Outcome (maximum 5 stars)	Total score (maximum 10)
Flores-Mir et al. 2018 [13]	4	1	3	8

Newcastle-Ottawa Quality Assessment Scale (adapted for cross sectional studies)

Selection (maximum 5 stars):

1. Representativeness of the sample:

a)Truly representative of the average in the target population. * (all subjects or random sampling)

b)Somewhat representative of the average in the target population. * (nonrandom sampling)

c)Selected group of users.

d)No description of the sampling strategy.

2. Sample size:

a) Justified and satisfactory. *

b) Not justified.

3. Non-respondents:

a) Comparability between respondents and non-respondents characteristics is established, and the response rate is satisfactory. *

b) The response rate is unsatisfactory, or the comparability between respondents and non-respondents is unsatisfactory.

c) No description of the response rate or the characteristics of the responders and the non-responders.

4. Ascertainment of the exposure (risk factor):

a) Validated measurement tool. **

b) Non-validated measurement tool, but the tool is available or described.*

c) No description of the measurement tool.

Comparability (maximum 2 stars):

1. The subjects in different outcome groups are comparable, based on the study design or analysis. Confounding factors are controlled.

a) The study controls for the most important factor (select one). *

b) The study control for any additional factor. *

Outcome (maximum 3 stars):

1. Assessment of the outcome:

a) Independent blind assessment. **

b) Record linkage. **

c) Self-report. *

d) No description.

2. Statistical test:

a) The statistical test used to analyze the data is clearly described and appropriate, and the measurement of the association is presented, including confidence intervals and the probability level (*p* value). *

b) The statistical test is not appropriate, not described or incomplete

For the evaluation of RoB for the non-randomized clinical trials, the ROBINS-I-tool [22] was used. The evaluated criteria were divided into pre-intervention, intervention, and post-intervention categories. The RoB was individually analyzed for each study and classified as low, moderate, serious, critical, and no information (Table 3).

**Table 3:** Risk of bias of included articles

Domain bias	Description
Pre-intervention	
Bias due to confounding	Assessment of baseline in of certain number of participants by age and malocclusion description Assessment of the method of pain evaluation Assessment of time of evaluation
Bias in selection of participants into the study	Assessment of participants eligibility criteria Evaluation of eligible participants exclusion or difference between the follow-up period
Intervention	
Bias in classification of interventions	Assessment of the intervention status—use of the aligner was not properly described (change of the aligner) Use of additional orthodontic methods to correct malocclusion (ex: MI, elastic) Use of analgesic
Post-intervention	
Bias due to deviations from intended interventions	Evaluation of the systematic differences between the intervention (group that used the aligner) and the comparison group when there is no information about the evaluation of the pain Use of analgesic for pain relief during orthodontic treatment
Bias due to missing data	In the event of loss of follow-up, incomplete collection of data and exclusion of participants from the analysis
Bias in measurement of the outcomes	When assessments of pain perception were not reported or were measured with error When not all the measures established in the different treatment times are presented When the use of analgesic is mentioned or not
Bias in selection of the reported results	Selective report of results when the effect of all measurements of results has not been fully reported

For the randomized clinical trial, the RoB was performed using Cochrane Collaboration RoB 2.0 tool [23], analyzing six domains: random sequence generation, allocation concealment, blinding of patients and personnel, blinding of outcome assessor, incomplete outcome data, and selective outcome reporting (Table 4).

**Table 4:** Risk of bias of included studies

	Risk of bias					
Study	Random sequence generation	Allocation concealment	Blinding of patients, personnel	Blinding of outcome assessor	Incomplete outcome data	Selective outcome reporting
White et al. (2017) [2]	Low	Low	Low	Low	Low	Low

### Summary measures

Clinical heterogeneity was measured assessing the treatment protocol, according to the archwire sequences and use of the aligners, times of evaluation of pain levels, use of analgesics during orthodontic treatment, different prescriptions of the fixed appliances, and other outcomes such as soft tissue irritation and eating disorders. The assessment of pain levels was evaluated through a visual analog scale (VAS).

For continuous outcomes, descriptive statistics, such as mean differences and standard deviations, were used to summarize the data from the included studies.

### RoB/quality assessment among studies

The quality of evidence of the included studies was made according to The Grading of Recommendations Assessment, Development and Evaluation (GRADEpro Guideline Development Tool, available online at gradepro.org) [24].

### Synthesis of results

A meta-analysis was not justifiable because of the large amount of clinical, statistical, and methodological heterogeneity.

## Results

### Study selection and characteristics

**Fig. 1:**
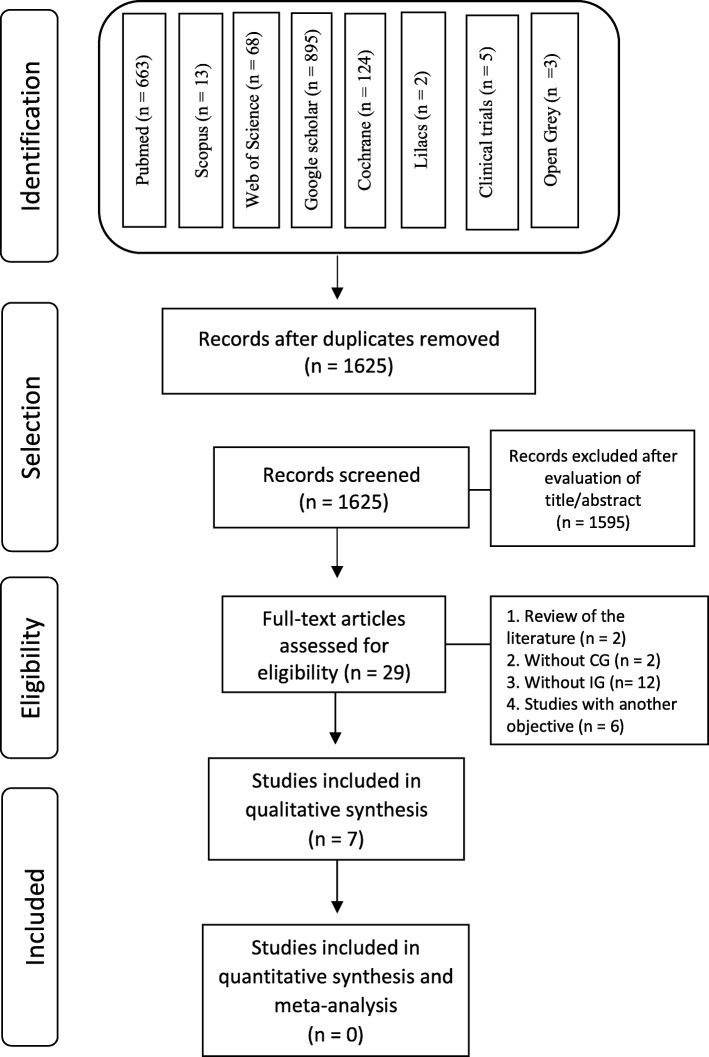
Diagram with number of records at each stage of the review according to PRISMA statement

A total of 1773 studies were identified in the following databases: PubMed (663), Cochrane (124), Web of Science (68), Scopus (13), Lilacs (2), Google Scholar (895) Clinical Trials (5), and OpenGrey (3). A manual search was also undertaken but no articles were found. The identified articles were exported to the Mendeley Desktop (version 1.19.4) reference manager to remove duplicates, and a total of 1625 articles remained after the duplicates were removed. A flow diagram of the process of identification, inclusion, and exclusion of studies is presented in Fig. 1.

The exclusion of articles by title and abstract was done by two evaluators (PC and DG), and in the end, 29 studies were selected to be evaluated by full text. Of these, 22 were excluded because 2 were a literature review, 14 did not have a control or an intervention group, 3 failed to evaluate pain, and 3 were not related to the objectives of this systematic review (Table 5).

**Table 5:** List of excluded studies (with reason)

Reference	Reason for exclusion
Abu Alhaija et al. (2015)	No intervention group
Ashkenazi et al. (2014)	No intervention group
Bergius et al. (2000)	Literature review
Bretz et al. (2018)	No intervention group
Caniklioglu et al. (2004)	No intervention group
Djeu et al (2014)	No pain evaluation
Fetouh (2008)	No pain evaluation
Fleming et al. (2009)	No intervention group
Kavaliauskiene et al. (2012)	No intervention group
Ke et al. (2019)	No pain evaluation
Kim (2013)	No control group
Mai-Tam (2018)	Literature review
Maldotti et al. (2013)	No control group
Noll et al. (2017)	Study not related with the SR objective
Pacheco-Pereira et al. (2015)	Study not related with the SR objective
Phuong (2018)	Study not related with the SR objective
Polat (2007)	No intervention group
Rakhshan (2015)	No intervention group
Salcedo-Bugarín (2018)	No intervention group
Scheurer et al. (1996)	No intervention group
Sergl et al. (1998)	No intervention group
Zealaiy et al. (2018)	No intervention group

*SR* systematic review

A total of seven studies were finally included. Five were prospective non-randomized clinical studies [10–12, 20, 25], one was a cross-sectional study [13], and one was a randomized clinical trial [2]. The mean age of the control group (group with fixed appliances) ranged between 23.56 [10] and 28.6 [11], but four studies [2, 12, 13, 25] did not report this information. A homogeneity was observed due the type of aligner used in all studies (Invisalign aligner); however, different types of fixed appliances were used as a control group, such as Edgewise [20], Damon Q (Ormco, Orange, CA) [10, 25] (Ormco, Glendora, CA), Speed [25] (Strite Industries Ltd., Ontario, Canada), Radiance (American Orthodontics, Sheboygan, WI) in the maxillary arch, and Alexander (American Orthodontics, Sheboygan, WI) in the mandibular arch [2]. One study just reported a use of a straight-wire appliance from GAC or Ormco [12], and two studies did not report any information [11, 13].

In five studies [2, 10, 20, 25, 26], a VAS was used as a method for evaluating pain level, one study used a questionnaire at the end of treatment [13], and two studies used both methods [11, 12].

When evaluating follow-up time, six studies [2, 10–12, 20, 25] reported daily evaluations during 1 week until 3 months of follow-up, and only one study evaluated pain level at the end of treatment [13].

### RoB within studies

The Newcastle-Ottawa scale for cross-sectional studies was applied for one study [13] and was classified with a good quality of evidence. A lower grade was applied for the selection domain due to the representativeness of the sample that was ranked as selected groups of users.

The ROBINS-I-Tool (Risk of Bias in Non-randomized Studies-of Interventions) was used in five studies [10–12, 20, 25] (Table 1). Reasons related to increased RoB included confusing information (description of the malocclusion, method of pain evaluation, and follow-up time). Only one [10] study presented a low RoB and four [11, 12, 20, 25] showed a moderate RoB (Table 6). The major reason for this grading was related to the use of analgesics and this information was not properly reported (use or not). One study [11] was classified as high RoB in the intervention domain due to bias in classification of interventions, which included the assessment of the intervention status, use of additional orthodontic methods to correct, and use of analgesic.

**Table 6:** Risk of bias of the included studies, according to the ROBINS-I tool

Domains								
	Pre-intervention		Intervention	Post-intervention				
Author	Bias due to confounding	Bias in selection of participants for the study	Bias in classifying interventions	Bias due to deviations from intended interventions	Bias due to missing data	Bias to measuring outcomes	Bias in selecting reported results	Overall risk of bias judgment
Almasoud (2018)	Low	Low	Low	Moderate	Low	Low	Low	Moderate
Fujiyama et al. (2014)	Moderate	Low	Moderate	Low	Low	Low	Moderate	Moderate
Mais-Damois et al. (2015)	Moderate	Low	Low	Moderate	Low	Low	Moderate	Moderate
Miller et al. (2007)	Moderate	High	High	Moderate	Low	Low	Low	High
Shalish et al. (2007)	Moderate	Moderate	Moderate	Moderate	Low	Low	Low	Moderate

For the randomized clinical trial [2], RoB was evaluated according to the Cochrane Collaboration RoB 2.0 tool, which presented a low RoB in all domains: random sequence generation, allocation concealment, blinding of patients and personnel, blinding of outcome assessor, incomplete outcome data, and selective outcome reporting (Table 3).

### Summary of individual studies’ results

Among all included studies [2, 10–13, 20, 25], pain scores were obtained 24 h after the beginning of treatment, and four included articles reported higher pain levels for fixed appliances during this period. However, only one investigation [10] found a statistically significant difference. Two others studies [2, 11] only reported a significant difference on day 3 and on day 4. Both studies reported that pain levels were higher in the group treated with fixed appliances. During days 5–7, only one study [2] observed a significantly higher level of pain in the patients with fixed appliances, but the highest level of pain was on the third day. Two studies [12, 25] evaluated pain on day 14 and reported no significant differences (*p* > 0.05) in pain level between groups. Only one study [2] performed this evaluation 2 months after starting treatment, and significant differences were found only on day 1 (*p* = 0.045) and day 2 (*p* = 0.041), with higher levels of pain in the control groups.

One study [25] compared different prescriptions of self-ligating appliances, Speed vs Damon, with Invisalign. Statistical differences were found between the Speed and Invisalign prescription only in the first activation, .016″ NiTi versus first aligner, and in the fourth phase, .019″ × .025″ CuNiTi and tenth aligner, 3 days after a follow-up appliance. In these two evaluations, the group that used a fixed appliance presented higher levels of pain when compared to the Invisalign group. Although one paper [12] reported a higher pain level for the aligner group for all evaluation times, 24 h and 14 days, no statistically significant (*p* > 0.05) difference was found for any time point.

Five studies [2, 10–12, 25] reported the use of analgesics, of which three studies found statistical differences in time points for 4 h [10] (*p* = 0.001), 24 h [10, 25] (*p* = 0.001 and *p* = 0.025), day 2 [2, 25] (*p* = 0.0023 and *p* < 0.05), and day 3 [11] (*p* = 0.006), and in all these cases, patients treated with fixed appliances reported a higher analgesic consumption. Only one study [12] observed that analgesic use was higher in the Invisalign group, since they discontinued their use on day 6, which was different from the control group that stopped their use on day 4. One study [13] also assessed QoL and patient satisfaction during orthodontic treatment, finding a statistical difference only in the evaluation of eating and chewing, where the Invisalign group presented a better response than the control group (47% and 24%, respectively).

Soft tissue irritation was reported to be lower in the Invisalign group in two studies [12, 25] as well as the assessments related to eating disorders [12].

### Certainty level

The quality of the articles was assessed using the GRADE system described in Table 7. All timepoints evaluated in the studies were rated with low certainty of evidence in all CCT studies [10–12, 20, 25], except for the RCT [2] that was rated with high certainty of evidence. Just one study [13] was not included in the evaluation because it was a cross-sectional study and had not made timepoint evaluations.

**Table 7:** Grading system according to GRADEpro

Clear aligners compared to fixed appliances for pain											
Certainty assessment							Summary of findings				
No. of participants (studies) followed up	Risk of bias	Inconsistency	Indirectness	Imprecision	Publication bias	Overall certainty of evidence	Study event rates (%)		Relative effect (95% CI)	Anticipated absolute effects	
							With fixed appliances	With clear aligners		Risk with fixed appliances	Risk difference with clear aligners
1st, 3rd, and 7th day (follow up: mean 1 days; assessed with VAS scale)											
336 (5 non-randomized studies)	Serious^a^	Serious^a^	Not serious	Very serious^b^	All plausible residual confounding would reduce the demonstrated effect dose response gradient	⨁⨁◯◯ low	181 participants	155 participants	Not estimable	Low	
										0 per 1.000	
2nd, 4th, 5th, and 6th day (follow up: mean 1 days; assessed with VAS score)											
234 (3 non-randomized studies)	Serious^a^	Serious^a^	Not serious	Very serious	All plausible residual confounding would reduce the demonstrated effect dose response gradient	⨁⨁◯◯ low	110 participants	124 participants	Not estimable	Low	
										0 per 1.000	
14th day (follow up: mean 1 days; assessed with VAS score)											
119 (2 non-randomized studies)	Serious ^c^	Serious ^c^	Not serious	Very serious^b^	All plausible residual confounding would reduce the demonstrated effect dose response gradient	⨁⨁◯◯ low	67 participants	52 participants	Not estimable	Low	
										0 per 1.000	
21st, 22nd, 23rd, 36th, and 37th day (follow up: mean 1 days; assessed with VAS score)											
93 (1 non-randomized study)	Not serious	Very serious^d^	Not serious	Very serious^b^	All plausible residual confounding would reduce the demonstrated effect dose response gradient	⨁⨁◯◯ low	55 participants	38 participants	Not estimable	Low	
										0 per 1.000	
24th, 25th, 26th, 27th, 35th, 38th, 39th 40th, and 41st day (follow up: mean 1 days; assessed with VAS score)											
93 (1 non-randomized study)	Not serious	Very serious^d^	Not serious	Very serious^b^	All plausible residual confounding would reduce the demonstrated effect dose response gradient	⨁⨁◯◯ low	55 participants	38 participants	Not estimable	Low	
										0 per 1.000	
Baseline (follow up: mean 1 days; assessed with VAS SCORE)											
223 (3 non-randomized studies)	Serious^a^	Serious^a,c^	Not serious	Very serious ^b^	All plausible residual confounding would reduce the demonstrated effect dose response gradient	⨁⨁◯◯ low	121 participants	102 participants	Not estimable	Low	
										0 per 1.000	
Baseline, 1st, 2nd, 14th, 30th, 33rd, 34th, 60th, 61st, 62nd, 63rd, and 64th day (follow up: mean 1 days; assessed with VAS score)											
41 (1 RCT)	Not serious	Not serious	Not serious	Very serious^b^	All plausible residual confounding would reduce the demonstrated effect dose response gradient	⨁⨁⨁⨁ high	18 participants	23 participants	Not estimable	Low	
										0 per 1.000	
3rd, 4th, 5th, 6th, 7th, 31st, and 32nd day (follow up: mean 1 days; assessed with VAS score)											
41 (1 RCT)	Not serious	Not serious	Not serious	Very serious^b^	All plausible residual confounding would reduce the demonstrated effect dose response gradient	⨁⨁⨁⨁ high	18 participants	23 participants	Not estimable	Low	
										0 per 1.000	

*CI* confidence interval

^a^This will down grade because one article was classified with a serious RoB

^b^This will downgrade because the use of analgesic was not properly described and it may mask the real pain reported by the patients

^c^This will downgrade because two articles were classified with moderate RoB

^d^This will downgrade because one article as classified with a moderate RoB

### Synthesis of results and additional analyses

It was not possible to perform a meta-analysis because of large amount of clinical, methodological, and statistical heterogeneity among the included studies, mainly due to differences between the archwire sequence in the fixed appliances and times of change for the aligners. In addition, attempts were made to contact the authors by email to collect missing data; however, only two of them responded, and they sent all the data available. Additional information still was not useful enough to justify a meta-analysis.

## Discussion

In recent years, continuous search for esthetic alternatives and comfortable orthodontic treatment approaches have been reasons for significant increases in the number of cases treated with clear aligners. Recent studies have shown that patients specifically treated with Invisalign were satisfied with their esthetic results and showed an improvement in their QoL, especially when related to their smile and during chewing and eating functions analyzed after treatment [13, 27]. However, concerning the efficacy of treatment, recent systematic reviews have suggested that this treatment modality presents some difficulties on specific orthodontic movements when compared with fixed appliances such as in rotation and vertical movements [14], ideal occlusal contacts, torque control, increasing transverse width and retention [16]. In addition, a study that evaluated the results of treatments performed with Invisalign and conventional brackets according to the American Board of Orthodontics’ objective classification system showed that treatment with fixed appliances are relatively superior than the treatment performed with Invisalign [28].

Despite the fact that fixed appliances have been the most effective traditional method for orthodontic treatment for many years and have shown good treatment efficiency, several studies have reported the negative side effects of this technique, especially plaque accumulation and difficulty of oral hygiene [26, 29]. Another important aspect commonly observed is pain experience and discomfort during orthodontic treatment [30] since 91–95% of patients experience some level of pain at different stages of treatment [8].

Pain is provoked by noxious stimuli and is a complex experience [30]. Therefore, it is important to understand the pain pattern during orthodontic treatment because pain and discomfort are two of the main reasons that affect the patient’s QoL during treatment [31]. In addition, fear of pain is one of the main reasons for discouraging orthodontic treatment [32] and previous studies have found that 8% [33] to 30% [34] of patients discontinue orthodontic treatment due to pain experienced at the early stages of treatment.

Four studies [10, 11, 13, 25] reported higher levels of pain for the group treated with fixed appliances during the first 24 h after beginning treatment, which corroborates with other studies [6, 35, 36], which show that the highest levels of pain are found 1 day after inserting initial archwires. Furthermore, the literature also shows that the pain is more intense during the first 3 days and is slowly minimized or disappears on the seventh day. This is in agreement with most of the included studies of this systematic review [2, 10, 11, 20]. This pattern of pain occurs due to initial orthodontic forces that cause discomfort due to compression of the periodontal ligament, leading to ischemia, edema [37], and release of inflammatory mediators during the first 24–48 h [38]. These mediators such as prostaglandins (e.g., PgE) and interleukins (e.g., IL-1β) sensitize nociceptors of the periodontal ligament, increasing discomfort. The levels of these mediators found in the gingival cervical fluid peak 24 h after the onset of orthodontic force and return to the reference values after 7 days [39]. This explains the pattern of pain observed during the first week after the application of orthodontic force.

Although the periods of higher and lower pain levels were similar for the fixed and Invisalign treated groups, in the present systematic review only one study [12] showed higher levels of pain for the group treated with aligners. They reported that this result may have been found due to a greater mechanical force applied at the beginning of Invisalign treatment; however, the sequence, time of use, and period of exchange of the aligners were not described.

Understanding that pain can affect the QoL of the individual, which can lead to worsening oral hygiene and have a psychosocial impact [40], many patients use analgesics for pain relief caused by orthodontic treatment. In the present systematic review, five studies reported the use of analgesics [2, 10–12, 25], and in all of them, the use of analgesics was similar to the periods of higher and lower pain levels. The perception of orthodontic pain is due to changes in blood flow caused by the appliances, and the use of analgesic may reduce the inflammatory process, consequently reducing the pain levels [41], although the use of these pain relief medications may mask the real pain reported by the patients leading to an uncertain result. Medication intake was higher in the fixed appliance group than in the Invisalign group as previously reported in the literature [8, 35]. The fact that patients with fixed appliances take more medications may underestimate the pain reported by them when treated with this type of appliance.

However, pain is a subjective process and can be influenced by several factors. Studies show that pain may be related to the individual’s personality and that patients who have some knowledge about orthodontic treatment and have more positive attitudes presented lower levels of pain during treatment [42, 43]. Therefore, it is suggested that the professionals inform the patients of any discomfort that may occur during orthodontic treatment and guide ways to alleviate it [42].

Knowing that the activation of the fixed appliance is done once a month and the aligners changed every 15 days, it may be reasonable to think that patients treated with aligners report lower pain levels at each activation, but it is felt for a longer period of time. That said, it is important to point out that few studies have evaluated pain over a longer period of treatment. A randomized clinical trial [2] performed this evaluation for 2 months and observed the pain in the subsequent appointments was lower in both groups. In the second month of maintenance, no statistical difference was observed.

The types of archwires should be taken in account since they have differences in some mechanical properties such as low elasticity module and coefficient of attrition, high resilience, flexibility and elastic recovery, and biocompatibility that are important characteristics to stimulate the adequate tissue response [44–46]. A laboratory study demonstrates that nickel-titanium archwire with addition of copper (CuNiTi) presented less favorable biologically deactivation loads compared to the other thermoactivated NiTi [47] which is consistent with a systematic review and meta-analyses [48] that found that patients treated with CuNiTi archwires presented greater levels of pain in the Likert scale than those patients treated with NiTi.

However, lower levels of pain found in patients treated with Invisalign may be related to the fact that removable appliances produce less tension, pressure, sensitivity, and pain than fixed appliances [49]. This reduced discomfort in clear aligners may be associated with proinflammatory mediators such as IL-1β because in the short term, these mediators increase sensitization by triggering receptor-associated kinases and ion channels, and in the long term, they persuade the transcriptional upregulation of receptors, leading to hyperalgesia [50]. So, it is reasonable to state that removable appliances predisposed to painless responses due to the intermittent forces when compared to the continuous forces of the fixed appliances [51]. Furthermore, they can be removed by the patients themselves for pain relief. In addition, it was hypothesized by one study [12] that these results among non-randomized investigations should be evaluated with caution since cases treated with Invisalign usually have lower rates of irregularity index, and this difference may influence the patient’s perception of pain, which is considered an important bias in the interpretation of the results. In this systematic review, only two studies [2, 10] considered crowding level as inclusion criteria, and in both of them, they range from mild to moderate. However, the other five studies [11–13, 20, 25] did not describe any information, and none of the included studies reported any differences in irregularity index between the evaluated groups. Despite that, there are controversial results about the correlation between the irregularity index and the perception of pain. Some studies found that there is no correlation [52–54], but a recent one found that crowding is a risk factor, and with each increase in crowding, there is a 1.10 times increase in painful sensation [55].

Another relevant factor is the type of malocclusion included in the studies. Some studies did not report inclusion criteria adequately [11–13, 20], and among those who reported [2, 10, 25], all included patients with mild or moderate malocclusion, Angle Class I malocclusion, and crowding of up to 5 mm, which may bias the results, since the more severe the malocclusion, the more it is related to the psychosocial well-being of the patient in pain-related scales, psychological discomfort, and social problems [56].

Overall, the present systematic review showed lower pain levels for the groups treated with Invisalign during the first days of treatment. The studies presented a high methodological quality according to the grading system, with the RoB varying from moderate [12, 20, 25] to high in five studies [11, 13], and only two [2, 10] of the studies presented a low RoB. Pain is one of many considerations, and predictability and technical outcome are more important, mainly considering that the difference does not seem to occur after the first months of the orthodontic treatment.

## Limitations

There is a high level of heterogeneity in the design of the studies included in this systematic review. Among these studies, we observed a great variation in relation to the types of fixed appliance used, and five different types were externally funded by companies. In addition, the sequence of the archwires used and the set of the aligner was poorly detailed. Both factors can strongly affect the results found in this systematic review.

Selection of the participants was only randomized in one study [2] that presented a high certainty of evidence. In all other studies [10–12, 20, 25], that were classified with low certainty of evidence, selection was done according to the order of appearance of patients seeking orthodontic treatment, and in some cases, the patient chose which type of device they wanted to be treated with.

In addition, the use of analgesics was not reported in all studies. This may be likely a significant confounding factor since it is well established in the literature that the use of this drug camouflages the actual levels of pain produced during orthodontic treatment.

No other clear aligner appliances were studied in the included studies. No conclusions/suggestions can therefore be made about other alternatives.

## Conclusion

Orthodontic patients treated with Invisalign appear to report lower levels of pain than those treated with fixed appliances during the first few days of treatment. However, the type of malocclusions was not comprehensively described which may lead to controversial results. Thereafter (up to 3 months), differences were not noted. Malocclusion complexity level among included studies was mild.

Based on the level of certainty, the results should be evaluated with caution, and it is suggested that studies with better methodological qualities be performed.

## Data Availability

The datasets used and/or analyzed during the current study are available from the corresponding author on reasonable request.

## Appendix

**Table 8:** Database and Search Strategy

Database	Search Strategy
Pubmed	Search ((((((((((Orthodontic Appliances[MeSH Terms]) OR Orthodontic Appliances[Title/Abstract]) OR Appliance, Orthodontic[Title/Abstract]) AND Appliances, Orthodontic[Title/Abstract]) OR Orthodontic Appliance[Title/Abstract])) OR (((((((((((((((((((((((((((((Orthodontic Appliances, Fixed[MeSH Terms]) OR Orthodontic Appliances, Fixed[Title/Abstract]) OR Appliance, Fixed Orthodontic[Title/Abstract]) OR Appliances, Fixed Orthodontic[Title/Abstract]) OR Fixed Orthodontic Appliance[Title/Abstract]) OR Fixed Orthodontic Appliances[Title/Abstract]) OR Orthodontic Appliance, Fixed[Title/Abstract]) OR Fixed Functional Appliances[Title/Abstract]) OR Appliance, Fixed Functional[Title/Abstract]) OR Appliances, Fixed Functional[Title/Abstract]) OR Fixed Functional Appliance[Title/Abstract]) OR Functional Appliance, Fixed[Title/Abstract]) OR Functional Appliances, Fixed[Title/Abstract]) OR Fixed Retainer[Title/Abstract]) OR Fixed Retainers[Title/Abstract]) OR Retainer, Fixed[Title/Abstract]) OR Retainers, Fixed[Title/Abstract]) OR Bonded Retainer[Title/Abstract]) OR Bonded Retainers[Title/Abstract]) OR Retainer, Bonded[Title/Abstract]) OR Retainers, Bonded[Title/Abstract]) OR Fixed Appliances[Title/Abstract]) OR Appliance, Fixed[Title/Abstract]) OR Appliances, Fixed[Title/Abstract]) OR Fixed Appliance[Title/Abstract]) OR Permanent Retainer[Title/Abstract]) OR Permanent Retainers[Title/Abstract]) OR Retainer, Permanent[Title/Abstract]) OR Retainers, Permanent[Title/Abstract])) OR ((((((((((((((((((((((((((Orthodontic Appliances, Removable[MeSH Terms]) OR Orthodontic Appliances, Removable[Title/Abstract]) OR Appliance, Removable Orthodontic[Title/Abstract]) OR Appliances, Removable Orthodontic[Title/Abstract]) OR Orthodontic Appliance, Removable[Title/Abstract]) OR Removable Orthodontic Appliance[Title/Abstract]) OR Removable Orthodontic Appliances[Title/Abstract]) OR Clear Dental Braces[Title/Abstract]) OR Brace, Clear Dental[Title/Abstract]) OR Braces, Clear Dental[Title/Abstract]) OR Clear Dental Brace[Title/Abstract]) OR Dental Brace, Clear[Title/Abstract]) OR Dental Braces, Clear[Title/Abstract]) OR Clear Aligner Appliances[Title/Abstract]) OR Aligner Appliance, Clear[Title/Abstract]) OR Aligner Appliances, Clear[Title/Abstract]) OR Appliance, Clear Aligner[Title/Abstract]) OR Appliances, Clear Aligner[Title/Abstract]) OR Clean Dental Braces[Title/Abstract]) OR Brace, Clean Dental[Title/Abstract]) OR Braces, Clean Dental[Title/Abstract]) OR Clean Dental Brace[Title/Abstract]) OR Dental Brace, Clean[Title/Abstract]) OR Dental Braces, Clean[Title/Abstract]) OR Invisalign[Title/Abstract]) OR Invisaligns[Title/Abstract])) OR (((Traditional Brackets[Title/Abstract]) OR Edgewise appliance[Title/Abstract]) OR Passive self-ligating[Title/Abstract]))) AND (((((((((Pain Perception[MeSH Terms]) OR Pain Perception[Title/Abstract]) OR Pain Perceptions[Title/Abstract]) OR Perception, Pain[Title/Abstract]) OR Perceptions, Pain[Title/Abstract])) OR (((((((((((((((((((((((((((((((((((((((((((((((Pain Measurement[MeSH Terms]) OR Pain Measurement[Title/Abstract]) OR Measurement, Pain[Title/Abstract]) OR Measurements, Pain[Title/Abstract]) OR Pain Measurements[Title/Abstract]) OR Assessment, Pain[Title/Abstract]) OR Assessments, Pain[Title/Abstract]) OR Pain Assessments[Title/Abstract]) OR Pain Assessment[Title/Abstract]) OR Analgesia Tests[Title/Abstract]) OR Analgesia Test[Title/Abstract]) OR Test, Analgesia[Title/Abstract]) OR Tests, Analgesia[Title/Abstract]) OR Nociception Tests[Title/Abstract]) OR Nociception Test[Title/Abstract]) OR Test, Nociception[Title/Abstract]) OR Tests, Nociception[Title/Abstract]) OR McGill Pain Questionnaire[Title/Abstract]) OR Pain Questionnaire, McGill[Title/Abstract]) OR Questionnaire, McGill Pain[Title/Abstract]) OR McGill Pain Scale[Title/Abstract]) OR Pain Scale, McGill[Title/Abstract]) OR Scale, McGill Pain[Title/Abstract]) OR Visual Analog Pain Scale[Title/Abstract]) OR Visual Analogue Pain Scale[Title/Abstract]) OR Analogue Pain Scale[Title/Abstract]) OR Analogue Pain Scales[Title/Abstract]) OR Pain Scale, Analogue[Title/Abstract]) OR Pain Scales, Analogue[Title/Abstract]) OR Scale, Analogue Pain[Title/Abstract]) OR Scales, Analogue Pain[Title/Abstract]) OR Analog Pain Scale[Title/Abstract]) OR Analog Pain Scales[Title/Abstract]) OR Pain Scale, Analog[Title/Abstract]) OR Pain Scales, Analog[Title/Abstract]) OR Scale, Analog Pain[Title/Abstract]) OR Scales, Analog Pain[Title/Abstract]) OR Formalin Test[Title/Abstract]) OR Formalin Tests[Title/Abstract]) OR Test, Formalin[Title/Abstract]) OR Tests, Formalin[Title/Abstract]) OR Tourniquet Pain Test[Title/Abstract]) OR Pain Test, Tourniquet[Title/Abstract]) OR Pain Tests, Tourniquet[Title/Abstract]) OR Test, Tourniquet Pain[Title/Abstract]) OR Tests, Tourniquet Pain[Title/Abstract]) OR Tourniquet Pain Tests[Title/Abstract])) OR (((((Patient Comfort[MeSH Terms]) OR Patient Comfort[Title/Abstract]) OR Comfort, Patient[Title/Abstract]) OR Comfort Care[Title/Abstract]) OR Care, Comfort[Title/Abstract])) OR (((((Toothache[MeSH Terms]) OR Toothache[Title/Abstract]) OR Toothaches[Title/Abstract]) OR Odontalgia[Title/Abstract]) OR Odontalgias[Title/Abstract])) Sort by: Best Match
	Search ((((((((((Orthodontic Appliances[MeSH Terms]) OR Orthodontic Appliances[Title/Abstract]) OR Appliance, Orthodontic[Title/Abstract]) AND Appliances, Orthodontic[Title/Abstract]) OR Orthodontic Appliance[Title/Abstract])) OR (((((((((((((((((((((((((((((Orthodontic Appliances, Fixed[MeSH Terms]) OR Orthodontic Appliances, Fixed[Title/Abstract]) OR Appliance, Fixed Orthodontic[Title/Abstract]) OR Appliances, Fixed Orthodontic[Title/Abstract]) OR Fixed Orthodontic Appliance[Title/Abstract]) OR Fixed Orthodontic Appliances[Title/Abstract]) OR Orthodontic Appliance, Fixed[Title/Abstract]) OR Fixed Functional Appliances[Title/Abstract]) OR Appliance, Fixed Functional[Title/Abstract]) OR Appliances, Fixed Functional[Title/Abstract]) OR Fixed Functional Appliance[Title/Abstract]) OR Functional Appliance, Fixed[Title/Abstract]) OR Functional Appliances, Fixed[Title/Abstract]) OR Fixed Retainer[Title/Abstract]) OR Fixed Retainers[Title/Abstract]) OR Retainer, Fixed[Title/Abstract]) OR Retainers, Fixed[Title/Abstract]) OR Bonded Retainer[Title/Abstract]) OR Bonded Retainers[Title/Abstract]) OR Retainer, Bonded[Title/Abstract]) OR Retainers, Bonded[Title/Abstract]) OR Fixed Appliances[Title/Abstract]) OR Appliance, Fixed[Title/Abstract]) OR Appliances, Fixed[Title/Abstract]) OR Fixed Appliance[Title/Abstract]) OR Permanent Retainer[Title/Abstract]) OR Permanent Retainers[Title/Abstract]) OR Retainer, Permanent[Title/Abstract]) OR Retainers, Permanent[Title/Abstract])) OR ((((((((((((((((((((((((((Orthodontic Appliances, Removable[MeSH Terms]) OR Orthodontic Appliances, Removable[Title/Abstract]) OR Appliance, Removable Orthodontic[Title/Abstract]) OR Appliances, Removable Orthodontic[Title/Abstract]) OR Orthodontic Appliance, Removable[Title/Abstract]) OR Removable Orthodontic Appliance[Title/Abstract]) OR Removable Orthodontic Appliances[Title/Abstract]) OR Clear Dental Braces[Title/Abstract]) OR Brace, Clear Dental[Title/Abstract]) OR Braces, Clear Dental[Title/Abstract]) OR Clear Dental Brace[Title/Abstract]) OR Dental Brace, Clear[Title/Abstract]) OR Dental Braces, Clear[Title/Abstract]) OR Clear Aligner Appliances[Title/Abstract]) OR Aligner Appliance, Clear[Title/Abstract]) OR Aligner Appliances, Clear[Title/Abstract]) OR Appliance, Clear Aligner[Title/Abstract]) OR Appliances, Clear Aligner[Title/Abstract]) OR Clean Dental Braces[Title/Abstract]) OR Brace, Clean Dental[Title/Abstract]) OR Braces, Clean Dental[Title/Abstract]) OR Clean Dental Brace[Title/Abstract]) OR Dental Brace, Clean[Title/Abstract]) OR Dental Braces, Clean[Title/Abstract]) OR Invisalign[Title/Abstract]) OR Invisaligns[Title/Abstract])) OR (((Traditional Brackets[Title/Abstract]) OR Edgewise appliance[Title/Abstract]) OR Passive self-ligating[Title/Abstract]))) AND (((((((((Pain Perception[MeSH Terms]) OR Pain Perception[Title/Abstract]) OR Pain Perceptions[Title/Abstract]) OR Perception, Pain[Title/Abstract]) OR Perceptions, Pain[Title/Abstract])) OR (((((((((((((((((((((((((((((((((((((((((((((((Pain Measurement[MeSH Terms]) OR Pain Measurement[Title/Abstract]) OR Measurement, Pain[Title/Abstract]) OR Measurements, Pain[Title/Abstract]) OR Pain Measurements[Title/Abstract]) OR Assessment, Pain[Title/Abstract]) OR Assessments, Pain[Title/Abstract]) OR Pain Assessments[Title/Abstract]) OR Pain Assessment[Title/Abstract]) OR Analgesia Tests[Title/Abstract]) OR Analgesia Test[Title/Abstract]) OR Test, Analgesia[Title/Abstract]) OR Tests, Analgesia[Title/Abstract]) OR Nociception Tests[Title/Abstract]) OR Nociception Test[Title/Abstract]) OR Test, Nociception[Title/Abstract]) OR Tests, Nociception[Title/Abstract]) OR McGill Pain Questionnaire[Title/Abstract]) OR Pain Questionnaire, McGill[Title/Abstract]) OR Questionnaire, McGill Pain[Title/Abstract]) OR McGill Pain Scale[Title/Abstract]) OR Pain Scale, McGill[Title/Abstract]) OR Scale, McGill Pain[Title/Abstract]) OR Visual Analog Pain Scale[Title/Abstract]) OR Visual Analogue Pain Scale[Title/Abstract]) OR Analogue Pain Scale[Title/Abstract]) OR Analogue Pain Scales[Title/Abstract]) OR Pain Scale, Analogue[Title/Abstract]) OR Pain Scales, Analogue[Title/Abstract]) OR Scale, Analogue Pain[Title/Abstract]) OR Scales, Analogue Pain[Title/Abstract]) OR Analog Pain Scale[Title/Abstract]) OR Analog Pain Scales[Title/Abstract]) OR Pain Scale, Analog[Title/Abstract]) OR Pain Scales, Analog[Title/Abstract]) OR Scale, Analog Pain[Title/Abstract]) OR Scales, Analog Pain[Title/Abstract]) OR Formalin Test[Title/Abstract]) OR Formalin Tests[Title/Abstract]) OR Test, Formalin[Title/Abstract]) OR Tests, Formalin[Title/Abstract]) OR Tourniquet Pain Test[Title/Abstract]) OR Pain Test, Tourniquet[Title/Abstract]) OR Pain Tests, Tourniquet[Title/Abstract]) OR Test, Tourniquet Pain[Title/Abstract]) OR Tests, Tourniquet Pain[Title/Abstract]) OR Tourniquet Pain Tests[Title/Abstract])) OR (((((Patient Comfort[MeSH Terms]) OR Patient Comfort[Title/Abstract]) OR Comfort, Patient[Title/Abstract]) OR Comfort Care[Title/Abstract]) OR Care, Comfort[Title/Abstract])) OR (((((Toothache[MeSH Terms]) OR Toothache[Title/Abstract]) OR Toothaches[Title/Abstract]) OR Odontalgia[Title/Abstract]) OR Odontalgias[Title/Abstract]))
	Final search: #1 AND #2
Scopus	( ( ( TITLE-ABS-KEY ( orthodontic AND appliances* ) OR TITLE-ABS-KEY ( "Appliance, Orthodontic" ) OR TITLE-ABS-KEY ( "Appliances, Orthodontic" ) OR TITLE-ABS-KEY ( "Orthodontic Appliance" ) ) ) OR ( ( TITLE-ABS-KEY ( orthodontic AND appliances, AND fixed* ) OR TITLE-ABS-KEY ( "Appliance, Fixed Orthodontic" ) OR TITLE-ABS-KEY ( "Appliances, Fixed Orthodontic" ) OR TITLE-ABS-KEY ( "Fixed Orthodontic Appliance" ) OR TITLE-ABS-KEY ( "Fixed Orthodontic Appliances" ) OR TITLE-ABS-KEY ( "Orthodontic Appliance, Fixed" ) OR TITLE-ABS-KEY ( "Fixed Functional Appliances" ) OR TITLE-ABS-KEY ( "Appliance, Fixed Functional" ) OR TITLE-ABS-KEY ( "Appliances, Fixed Functional" ) OR TITLE-ABS-KEY ( "Fixed Functional Appliance" ) OR TITLE-ABS-KEY ( "Functional Appliance, Fixed" ) OR TITLE-ABS-KEY ( "Functional Appliances, Fixed" ) OR TITLE-ABS-KEY ( "Fixed Retainer" ) OR TITLE-ABS-KEY ( "Fixed Retainers" ) OR TITLE-ABS-KEY ( "Retainer, Fixed" ) OR TITLE-ABS-KEY ( "Retainers, Fixed" ) OR TITLE-ABS-KEY ( "Bonded Retainer" ) OR TITLE-ABS-KEY ( "Bonded Retainers" ) OR TITLE-ABS-KEY ( "Retainer, Bonded" ) OR TITLE-ABS-KEY ( "Retainers, Bonded" ) OR TITLE-ABS-KEY ( "Fixed Appliances" ) OR TITLE-ABS-KEY ( "Appliance, Fixed" ) OR TITLE-ABS-KEY ( "Appliances, Fixed" ) OR TITLE-ABS-KEY ( "Fixed Appliance" ) OR TITLE-ABS-KEY ( "Permanent Retainer" ) OR TITLE-ABS-KEY ( "Permanent Retainers" ) OR TITLE-ABS-KEY ( "Retainer, Permanent" ) OR TITLE-ABS-KEY ( "Retainers, Permanent" ) ) ) OR ( ( TITLE-ABS-KEY ( orthodontic AND appliances, AND removable* ) OR TITLE-ABS-KEY ( "Appliance, Removable Orthodontic" ) OR TITLE-ABS-KEY ( "Appliances, Removable Orthodontic" ) OR TITLE-ABS-KEY ( "Orthodontic Appliance, Removable" ) OR TITLE-ABS-KEY ( "Removable Orthodontic Appliance" ) OR TITLE-ABS-KEY ( "Removable Orthodontic Appliances" ) OR TITLE-ABS-KEY ( "Clear Dental Braces" ) OR TITLE-ABS-KEY ( "Brace, Clear Dental" ) OR TITLE-ABS-KEY ( "Braces, Clear Dental" ) OR TITLE-ABS-KEY ( "Clear Dental Brace" ) OR TITLE-ABS-KEY ( "Dental Brace, Clear" ) OR TITLE-ABS-KEY ( "Dental Braces, Clear" ) OR TITLE-ABS-KEY ( "Clear Aligner Appliances" ) OR TITLE-ABS-KEY ( "Aligner Appliance, Clear" ) OR TITLE-ABS-KEY ( "Aligner Appliances, Clear" ) OR TITLE-ABS-KEY ( "Appliance, Clear Aligner" ) OR TITLE-ABS-KEY ( "Appliances, Clear Aligner" ) OR TITLE-ABS-KEY ( "Clear Aligner Appliance" ) OR TITLE-ABS-KEY ( "Clean Dental Braces" ) OR TITLE-ABS-KEY ( "Brace, Clean Dental" ) OR TITLE-ABS-KEY ( "Braces, Clean Dental" ) OR TITLE-ABS-KEY ( "Clean Dental Brace" ) OR TITLE-ABS-KEY ( "Dental Brace, Clean" ) OR TITLE-ABS-KEY ( "Dental Braces, Clean" ) OR TITLE-ABS-KEY ( "Invisalign" ) OR TITLE-ABS-KEY ( "Invisaligns" ) ) ) ) AND ( TITLE-ABS-KEY ( "invisalign" ) ) AND ( TITLE-ABS-KEY ( "pain" ) )
Web of Science	( ( TITLE-ABS-KEY ( orthodontic AND appliances* ) OR TITLE-ABS-KEY ( "Appliance, Orthodontic" ) OR TITLE-ABS-KEY ( "Appliances, Orthodontic" ) OR TITLE-ABS-KEY ( "Orthodontic Appliance" ) ) ) OR ( ( TITLE-ABS-KEY ( orthodontic AND appliances, AND fixed* ) OR TITLE-ABS-KEY ( "Appliance, Fixed Orthodontic" ) OR TITLE-ABS-KEY ( "Appliances, Fixed Orthodontic" ) OR TITLE-ABS-KEY ( "Fixed Orthodontic Appliance" ) OR TITLE-ABS-KEY ( "Fixed Orthodontic Appliances" ) OR TITLE-ABS-KEY ( "Orthodontic Appliance, Fixed" ) OR TITLE-ABS-KEY ( "Fixed Functional Appliances" ) OR TITLE-ABS-KEY ( "Appliance, Fixed Functional" ) OR TITLE-ABS-KEY ( "Appliances, Fixed Functional" ) OR TITLE-ABS-KEY ( "Fixed Functional Appliance" ) OR TITLE-ABS-KEY ( "Functional Appliance, Fixed" ) OR TITLE-ABS-KEY ( "Functional Appliances, Fixed" ) OR TITLE-ABS-KEY ( "Fixed Retainer" ) OR TITLE-ABS-KEY ( "Fixed Retainers" ) OR TITLE-ABS-KEY ( "Retainer, Fixed" ) OR TITLE-ABS-KEY ( "Retainers, Fixed" ) OR TITLE-ABS-KEY ( "Bonded Retainer" ) OR TITLE-ABS-KEY ( "Bonded Retainers" ) OR TITLE-ABS-KEY ( "Retainer, Bonded" ) OR TITLE-ABS-KEY ( "Retainers, Bonded" ) OR TITLE-ABS-KEY ( "Fixed Appliances" ) OR TITLE-ABS-KEY ( "Appliance, Fixed" ) OR TITLE-ABS-KEY ( "Appliances, Fixed" ) OR TITLE-ABS-KEY ( "Fixed Appliance" ) OR TITLE-ABS-KEY ( "Permanent Retainer" ) OR TITLE-ABS-KEY ( "Permanent Retainers" ) OR TITLE-ABS-KEY ( "Retainer, Permanent" ) OR TITLE-ABS-KEY ( "Retainers, Permanent" ) ) ) OR ( ( TITLE-ABS-KEY ( orthodontic AND appliances, AND removable* ) OR TITLE-ABS-KEY ( "Appliance, Removable Orthodontic" ) OR TITLE-ABS-KEY ( "Appliances, Removable Orthodontic" ) OR TITLE-ABS-KEY ( "Orthodontic Appliance, Removable" )
The Cochrane Library	#1 (Malocclusion$):ti,ab,kw OR (“Malocclusions”):ti,ab,kw OR (“Tooth Crowding”):ti,ab,kw OR (“Crowding, Tooth”):ti,ab,kw OR (“Crowdings, Tooth”):ti,ab,kw (Word variations have been searched) #2 (Malocclusion$):ti,ab,kw OR (“Crossbite”):ti,ab,kw OR (“Crossbites”):ti,ab,kw OR (“Cross Bite”):ti,ab,kw OR (“Bite, Cross”):ti,ab,kw (Word variations have been searched) #3 (Malocclusion$):ti,ab,kw OR ("Bites, Cross"):ti,ab,kw OR (“Cross Bites”):ti,ab,kw OR (“Angle's Classification”):ti,ab,kw OR (”Angle Classification”):ti,ab,kw (Word variations have been searched) #4 (Malocclusion$):ti,ab,kw OR (“Angles Classification”):ti,ab,kw OR (“Classification, Angle's”):ti,ab,kw (Word variations have been searched) #5 #1 and #2 and #3 and #4 #6 (Adult$):ti,ab,kw OR (“Adults”):ti,ab,kw (Word variations have been searched) #7 (Orthodontic Appliances$):ti,ab,kw OR (“Appliance, Orthodontic”):ti,ab,kw OR (“Appliances, Orthodontic”):ti,ab,kw OR (“Orthodontic Appliance”):ti,ab,kw (Word variations have been searched) #8 (Orthodontic Appliances, Fixed$):ti,ab,kw OR (“Appliance, Fixed Orthodontic”):ti,ab,kw OR (“Appliances, Fixed Orthodontic”):ti,ab,kw OR (“Fixed Orthodontic Appliance”):ti,ab,kw OR (”Fixed Orthodontic Appliances”):ti,ab,kw (Word variations have been searched) #9 (Orthodontic Appliances, Fixed$):ti,ab,kw OR (“Orthodontic Appliance, Fixed”):ti,ab,kw OR (“Fixed Functional Appliances”):ti,ab,kw OR (“Appliance, Fixed Functional”):ti,ab,kw OR (“Appliances, Fixed Functional”):ti,ab,kw (Word variations have been searched) #10 (Orthodontic Appliances, Fixed$):ti,ab,kw OR (“Fixed Functional Appliance”):ti,ab,kw OR (“Functional Appliance, Fixed”):ti,ab,kw OR (“Functional Appliances, Fixed”):ti,ab,kw OR (“Fixed Retainer”):ti,ab,kw (Word variations have been searched) #11 (Orthodontic Appliances, Fixed$):ti,ab,kw OR (“Fixed Retainers”):ti,ab,kw OR (“Retainer, Fixed”):ti,ab,kw OR (“Retainers, Fixed”):ti,ab,kw OR (“Bonded Retainer”):ti,ab,kw (Word variations have been searched) #12 (Orthodontic Appliances, Fixed$):ti,ab,kw OR (“Bonded Retainers”):ti,ab,kw OR (“Retainer, Bonded”):ti,ab,kw OR (“Retainers, Bonded”):ti,ab,kw OR (“Fixed Appliances”):ti,ab,kw (Word variations have been searched) #13 (Orthodontic Appliances, Fixed$):ti,ab,kw OR (“Appliance, Fixed”):ti,ab,kw OR (“Appliances, Fixed”):ti,ab,kw OR (“Fixed Appliance”):ti,ab,kw OR (“Permanent Retainer”):ti,ab,kw (Word variations have been searched) #14 (Orthodontic Appliances, Fixed$):ti,ab,kw OR (“Permanent Retainers”):ti,ab,kw OR (“Retainer, Permanent”):ti,ab,kw OR (“Retainers, Permanent”):ti,ab,kw (Word variations have been searched) #15 #8 #9 #10 #11 #12 #13 #14 #16 (Orthodontic Appliances, Removable$):ti,ab,kw OR (“Appliance, Removable Orthodontic”):ti,ab,kw OR (“Appliances, Removable Orthodontic”):ti,ab,kw OR (“Orthodontic Appliance, Removable”):ti,ab,kw OR (“Removable Orthodontic Appliance”):ti,ab,kw (Word variations have been searched) #17 (Orthodontic Appliances, Removable$):ti,ab,kw OR (“Removable Orthodontic Appliances”):ti,ab,kw OR (“Clear Dental Braces”):ti,ab,kw OR (“Brace, Clear Dental”):ti,ab,kw OR (“Braces, Clear Dental”):ti,ab,kw (Word variations have been searched) #18 (Orthodontic Appliances, Removable$):ti,ab,kw OR (“Clear Dental Brace"):ti,ab,kw OR (“Dental Brace, Clear"):ti,ab,kw OR (“Dental Braces, Clear”):ti,ab,kw OR (“Clear Aligner Appliances”):ti,ab,kw (Word variations have been searched) #19 (Orthodontic Appliances, Removable$):ti,ab,kw OR (“Aligner Appliance, Clear”):ti,ab,kw OR (“Aligner Appliances, Clear”):ti,ab,kw OR (“Appliance, Clear Aligner”):ti,ab,kw OR (“Appliances, Clear Aligner”):ti,ab,kw (Word variations have been searched) #20 (Orthodontic Appliances, Removable$):ti,ab,kw OR (“Clean Dental Brace”):ti,ab,kw OR (“Dental Brace, Clean”):ti,ab,kw OR (“Dental Braces, Clean”):ti,ab,kw OR (“Invisalign”):ti,ab,kw (Word variations have been searched) #21 (Orthodontic Appliances, Removable$):ti,ab,kw OR (“Invisaligns”):ti,ab,kw (Word variations have been searched) #22 #16 #17 #18 #19 #20 #21 #23 (Pain Perception$):ti,ab,kw OR (“Pain Perceptions”):ti,ab,kw OR (“Perception, Pain”):ti,ab,kw OR (“Perceptions, Pain”):ti,ab,kw (Word variations have been searched) #24 (Pain Measurement$):ti,ab,kw OR (“Measurement, Pain”):ti,ab,kw OR (“Measurements, Pain”):ti,ab,kw OR (“Pain Measurements”):ti,ab,kw OR (“Assessment, Pain”):ti,ab,kw (Word variations have been searched) #25 (Pain Measurement$):ti,ab,kw OR (“Assessments, Pain”):ti,ab,kw OR (“Pain Assessments”):ti,ab,kw OR (“Pain Assessment”):ti,ab,kw OR (“Analgesia Tests”):ti,ab,kw (Word variations have been searched) #26 (Pain Measurement$):ti,ab,kw OR (“Analgesia Test”):ti,ab,kw OR (“Test, Analgesia”):ti,ab,kw OR (“Tests, Analgesia”):ti,ab,kw OR (“Nociception Tests”):ti,ab,kw (Word variations have been searched) #27 (Pain Measurement$):ti,ab,kw OR (“Nociception Test”):ti,ab,kw OR (“Test, Nociception”):ti,ab,kw OR (“Tests, Nociception”):ti,ab,kw OR (“McGill Pain Questionnaire”):ti,ab,kw (Word variations have been searched) #28 (Pain Measurement$):ti,ab,kw OR (“Pain Questionnaire, McGill”):ti,ab,kw OR (“Questionnaire, McGill Pain”):ti,ab,kw OR (“McGill Pain Scale”):ti,ab,kw OR (“Pain Scale, McGill”):ti,ab,kw (Word variations have been searched) #29 (Pain Measurement$):ti,ab,kw OR (“Scale, McGill Pain”):ti,ab,kw OR (“Visual Analog Pain Scale”):ti,ab,kw OR (“Visual Analogue Pain Scale”):ti,ab,kw OR (“Analogue Pain Scale”):ti,ab,kw (Word variations have been searched) #30 (Pain Measurement$):ti,ab,kw OR (“Analogue Pain Scales”):ti,ab,kw OR (“Pain Scale, Analogue”):ti,ab,kw OR (“Pain Scales, Analogue”):ti,ab,kw OR (“Scale, Analogue Pain”):ti,ab,kw (Word variations have been searched) #31 (Pain Measurement$):ti,ab,kw OR (“Scales, Analogue Pain”):ti,ab,kw OR (“Analog Pain Scale”):ti,ab,kw OR (“Analog Pain Scales”):ti,ab,kw OR (“Pain Scale, Analog”):ti,ab,kw (Word variations have been searched) #32 (Pain Measurement$):ti,ab,kw OR (“Pain Scales, Analog”):ti,ab,kw OR (“Scale, Analog Pain”):ti,ab,kw OR (“Scales, Analog Pain”):ti,ab,kw OR (“Formalin Test”):ti,ab,kw (Word variations have been searched) #33 (Pain Measurement$):ti,ab,kw OR (“Formalin Tests”):ti,ab,kw OR (“Test, Formalin”):ti,ab,kw OR (“Tests, Formalin”):ti,ab,kw OR (“Tourniquet Pain Test”):ti,ab,kw (Word variations have been searched) #34 (Pain Measurement$):ti,ab,kw OR (“Tourniquet Pain Tests”):ti,ab,kw (Word variations have been searched) #35 (Pain Measurement$):ti,ab,kw OR (“Pain Test, Tourniquet”):ti,ab,kw OR (“Pain Tests, Tourniquet”):ti,ab,kw OR (“Test, Tourniquet Pain”):ti,ab,kw OR (“Tests, Tourniquet Pain”):ti,ab,kw (Word variations have been searched) #36 #24 #25 #26 #27 #28 #29 #30 #31 #32 #33 #34 #35 #37 (Patient Comfort$):ti,ab,kw OR (“Comfort, Patient”):ti,ab,kw OR (“Comfort Care”):ti,ab,kw OR (“Care, Comfort”):ti,ab,kw (Word variations have been searched) #38 (Toothache$):ti,ab,kw OR (“Toothaches”):ti,ab,kw OR (“Odontalgia”):ti,ab,kw OR (“Odontalgias”):ti,ab,kw (Word variations have been searched) #39 #5 OR #6 #40 ("Traditional Brackets") OR (“Edgewise appliance”) OR (“Passive self-ligating”) (Word variations have been searched) #41 #7 OR #15 OR #22 OR #40 #42 #23 OR #36 OR # 37 OR #38 #43 #39 AND #41 AND #42 #44 #30 AND #41 #45 #39 AND #42 #46 #41 AND #42 #47 #6 AND #41 AND #42 #48 #15 OR #22 OR #40 #49 #39 AND #48 AND 42 #50 #48 AND #42 #51 "Invisalign" #52 #50 AND #51 #53 #48 OR #51 #54 #42 AND #53 #55 #23 OR #36 #56 #53 AND #55 #57 "discomfort" #58 #42 OR #57 #59 #53 AND #58
Lilacs	Invisalign AND pain
Open Grey	Invisalign
Google Scholar	Adult + malocclusion + Orthodontic Appliances, Fixed + Orthodontic Appliances, Removable + comparison + Pain Measurement + Discomfort
Clinical Trials	Invisalign
OpenGrey	Invisalign

## Abbreviations

CCT: Non-randomized clinical trial
GRADE: The Grading of Recommendations Assessment, Development and Evaluation
PRISMA: Preferred Reporting Items for Systematic Review and Meta-Analysis
QoL: Quality of life
RCT: Randomized clinical trial
RoB: Risk of bias
ROBINS-I-Tool: Risk of Bias in Non-randomized Studies-of Interventions
VAS: Visual analog scale
